# *Klebsiella pneumoniae* ST258 Negatively Regulates the Oxidative Burst in Human Neutrophils

**DOI:** 10.3389/fimmu.2019.00929

**Published:** 2019-04-26

**Authors:** Luis A. Castillo, Federico Birnberg-Weiss, Nahuel Rodriguez-Rodrigues, Daiana Martire-Greco, Fabiana Bigi, Veronica I. Landoni, Sonia A. Gomez, Gabriela C. Fernandez

**Affiliations:** ^1^Laboratorio de Fisiología de los Procesos Inflamatorios, Instituto de Medicina Experimental (IMEX)- Consejo Nacional de investigaciones Científicas y Tecnológicas (CONICET)/Academia Nacional de Medicina de Buenos Aires, Buenos Aires, Argentina; ^2^Instituto de Agrobiotecnología y Biología Molecular (IABIMO), Instituto Nacional de Tecnología Agropecuaria (INTA), Consejo Nacional de investigaciones Científicas y Tecnológicas (CONICET), Buenos Aires, Argentina; ^3^Servicio de Antimicrobianos, Instituto Nacional de Enfermedades Infecciosas Dr. Carlos G. Malbrán (INEI), Administración Nacional de Laboratorios e Institutos de Salud (ANLIS), Buenos Aires, Argentina

**Keywords:** immune evasion, *Klebsiella pneumoniae*, neutrophils, respiratory burst, LPS

## Abstract

The epidemic clone of *Klebsiella pneumoniae* (Kpn), sequence type 258 (ST258), carbapenamase producer (KPC), commonly infects hospitalized patients that are left with scarce therapeutic option since carbapenems are last resort antibiotics for life-threatening bacterial infections. To improve prevention and treatment, we should better understand the biology of Kpn KPC ST258 infections. Our hypothesis was that Kpn KPC ST258 evade the first line of defense of innate immunity, the polymorphonuclear neutrophil (PMN), by decreasing its functional response. Therefore, our aim was to evaluate how the ST258 Kpn clone affects PMN responses, focusing on the respiratory burst, compared to another opportunistic pathogen, *Escherichia coli* (Eco). We found that Kpn KPC ST258 was unable to trigger bactericidal responses as reactive oxygen species (ROS) generation and NETosis, compared to the high induction observed with Eco, but both bacterial strains were similarly phagocytized and cause increases in cell size and CD11b expression. The absence of ROS induction was also observed with other Kpn ST258 strains negative for KPC. These results reflect certain selectivity in terms of the functions that are triggered in PMN by Kpn, which seems to evade specifically those responses critical for bacterial survival. In this sense, bactericidal mechanisms evasion was associated with a higher survival of Kpn KPC ST258 compared to Eco. To investigate the mechanisms and molecules involved in ROS inhibition, we used bacterial extracts (BE) and found that BE were able to inhibit ROS generation triggered by the well-known ROS inducer, fMLP. A sequence of experiments led us to elucidate that the polysaccharide part of LPS was responsible for this inhibition, whereas lipid A mediated the other responses that were not affected by bacteria, such as cell size increase and CD11b up-regulation. In conclusion, we unraveled a mechanism of immune evasion of Kpn KPC ST258, which may contribute to design more effective strategies for the treatment of these multi-resistant bacterial infections.

## Introduction

*Klebsiella pneumoniae* (Kpn) is a Gram-negative pathogen causing a wide range of infections from urinary tract infections to pneumonia. Kpn is a member of the so-called ESKAPE group of microorganisms, a term that emphasizes the fact that they effectively “escape” the effects of antibacterial drugs (1). Antimicrobial resistance is a significant problem for the treatment of infectious diseases caused by resistant bacteria worldwide. Specifically, resistance to carbapenems, the antibiotics of last resort for life-threatening bacterial infections, has significantly increased mortality and morbidity in patients hospitalized in intensive care units or in long-term care facilities (2). As an example, mortality in patients suffering from bacteriemia or pulmonary infections caused by carbapenem-resistant Kpn strains ranges between ~30 and 70% (3). A clinically relevant Kpn clone has been genetically classified as multilocus sequence type 258 (ST258), which is a hyper-epidemic clone responsible for the global dispersion of carbapenem resistance. This resistance is conferred by an enzyme known as Kpn-Carbapenemase (KPC) (3), and the strains belonging to the ST258 are also resistant to all β-lactam antibiotics and generally contain additional resistance genes that confer resistance to aminoglycosides and quinolones (3). In Argentina, since 2010, the entry of ST258 into the country has altered our health system, since today this clone has been detected in most health institutions of the country (4). Although the ST258 clone is the most extended Kpn KPC lineage, the basis of its success, outside of antibiotic resistance, remains unknown.

In this sense, our hypothesis is that evasion strategies of the ST258 clone could allow it to escape the immune system. This could favor a rapid transmission and persistence within the community, and particularly, in the intra-hospital environment. In fact, some mechanisms of immune evasion in other not-ST258 not-KPC+ Kpn strains have been described, such as the resistance and down-regulation of β-defensins in the lung, the resistance to complement, and a reduction in their phagocytosis. These strategies have been associated with components of the polysaccharide capsule of Kpn, and to mucoviscosity phenotypes (5–8). In particular, the infection biology of Kpn KPC ST258 is poorly understood. The exact consequences of Kpn KPC ST258-PMN interaction need to be elucidated, in order to establish the success of KPC and to start delineating new possible therapeutic approaches.

PMN are the first cell line of antibacterial host defense. During the first steps of PMN activation, an increment in their forward scatter (FSC) flow cytometry parameter occurs. This has been associated with the spreading process itself, but also to the translocation and fusion of easily mobilized granules with the cytoplasmatic membrane (exocytosis), exposing the interior membrane surface of the granules to the exterior, therefore increasing the total surface area of the cell and, in consequence, the FSC (9, 10). Additionally, exocytosis causes the up-regulation in the plasmatic membrane of molecules and receptors involved in PMN adhesion to endothelial cells, such as CD11b.

Upon encountering bacteria, PMN capture, ingest, and kill them by the production of reactive oxygen species (ROS) within intracellular phagosomes (11). ROS are produced by a multicomponent oxidase complex, named the NADPH oxidase, which is unassembled and inactive in resting cells but assembles at the plasma or phagosomal membrane upon PMN activation. Critical elements of the oxidase components are segregated in the membrane and cytosol. Upon activation, these components translocate, in order to interact with the membrane components of the complex, where a functional oxidase is assembled and becomes active. Additionally, PMN were shown to generate web-like extracellular fibers known as neutrophil extracellular traps (NETs), composed of deoxyribonucleic acid (DNA), histones, and antimicrobial granule proteins, which are highly effective at trapping and killing invasive bacteria (12). NETosis is usually dependent on ROS generation, although ROS-independent NETosis has also been reported (13). Moreover, ROS-independent mechanisms are also important in PMN-mediated killing. These mechanisms include the delivery of proteolytic enzymes stored in PMN granules into the phagosome.

Considering the clinical relevance and rapid dissemination of Kpn KPC ST258, it is necessary to better understand the interaction PMN-bacteria in order to design new strategies that might contribute in the treatment of infections with this multi-resistant bacterial strain. Therefore, our aim was to study whether Kpn KPC ST258 is able to actively evade the first line of defense of the immune response, the PMN. For this purpose, we challenged human PMN with Kpn KPC ST258 and measured different PMN responses. For comparison, we used another Gram-negative opportunistic bacillus, *Escherichia coli* (Eco). Moreover, as we found that Kpn KPC ST258 was a poor inducer of ROS generation, the mechanisms and molecules involved in this phenomenon were also investigated.

## Materials and Methods

### Ethics Statement

Human normal samples were obtained from voluntary donors. This study was performed according to institutional guidelines (National Academy of Medicine, Buenos Aires, Argentina) and received the approval of the institutional ethics committee (No. 12524/17/X), and written informed consent was provided by all the subjects.

### Blood Samples

Blood samples were obtained from healthy volunteer donors who had not taken any medication for at least 10 days before the day of sampling. Blood was obtained by venipuncture of the forearm vein and was drawn directly into citrated plastic tubes.

### Polymorphonuclear Neutrophil (PMN) Isolation

Neutrophils were isolated by Ficoll-Hypaque gradient centrifugation (Ficoll Pharmacia, Uppsala; Hypaque, Wintthrop Products, Buenos Aires, Argentina) and dextran sedimentation, as previously described (14). Contaminating erythrocytes were removed by hypotonic lysis. After washing, the cells (96% neutrophils on May Grünwald/Giemsa-stained Cyto-preps) were suspended in RPMI 1640 supplemented with 2% heat-inactivated fetal calf serum (FCS) and used immediately after.

### Bacterial Strains and Cultures

The experiments were performed using two types of Gram-negative bacteria: *E. coli* (ATCC® 25922™) and a local clinical isolate of *Klebsiella pneumoniae* KPC carbapenemase producer, belonging to sequence type 258 (Kpn KPC ST258, Strain ID: M9885. See also Supplementary Material 1). Other bacterial strains used were: Kpn ATCC 700603 (not-ST258), three Kpn ST258 KPC negative (M19091, M19216, and M19145), and three Kpn ST258 KPC positive (M22738, M22810, and M22910) strains. For details see Supplementary Material 1. Bacteria were grown in Tryptic Soy Broth (TSB) (Britania) for 18 h at 37°C. 100 μL of the culture was added into 10 mL of fresh TSB, and grown for another 4 h with agitation, until the organism reached log phase. Bacteria was pelleted by centrifugation at 9,600 g for 15 min, washed twice in phosphate buffered saline (PBS) 1x, and resuspended at the desired concentration. Bacteria concentration was determined by measuring O.D. at 600 nm, and adjusting to 0.09 absorbance units, that is equivalent to 1 × 10^8^ colony forming units (CFU)/mL; CFU concentration was confirmed by counting on Tryptic Soy Agar (TSA).

### Recombinant Bacterial Strains Expressing Green Fluorescent Protein (GFP)

Fifty milliliters of Eco and Kpn KPC ST258 were cultured on LB until O.D. 600 nm = 0.6, harvested by centrifugation at 10,000 g for 10 min and washed four times with one volume of 10% glycerol and finally resuspended in 500 μl of 10% glycerol. These bacteria (50 μl) were electroporated with 1 μg of plasmid pML1335, kindly provided by Dr. Michael Niederweis, at 200 ohms, 2.5 V and 25 μFa. Plasmid pML1335 (15) contains the GFP gene under mycobacterial promotor (Psmyc). This vector carries ColE1 for replication and a hygromycin resistance gene. After electroporation bacteria were cultivated in 1 ml of SOC medium during 1 h and then plated on LB agar with hygromycin (200 μg/ml). Plates were incubated overnight at 37°C. The presence of GFP in individual colonies was evaluated by microscopy, irradiating the bacteria with ultraviolet light. Only colonies containing “green” bacteria were selected and used in further experiments.

### Phagocytosis Studies

#### Flow Cytometry

PMN (1 × 10^6^) were incubated with 1 × 10^7^ CFU of GFP-Kpn KPC ST258 or GFP-Eco for 1 h at 37°C (total bacteria-PMN interaction) or 4°C (for bacterial adhesion) in 5% CO_2_. After incubation, GFP+ PMN were evaluated by flow cytometry and expressed as the percentage of PMN associated to GFP-bacteria (37°C) or the percentage of PMN with bacteria attached (4°C). The percentage of phagocytosis was calculated subtracting the percentage of adhesion to the total interaction.

#### Confocal Laser Scanning Microscopy

PMN (3 × 10^5^) were mixed with 3 × 10^6^ CFU of GFP-Kpn KPC ST258 or GFP-Eco in cold RPMI with 2% FCS and seeded gently onto glass coverslips coated with 0.001% poly-L-lysine in a 24-well plate. Assay plates were centrifuged at 524 g during 5 min at 4°C to synchronize phagocytosis. Samples were incubated at 37°C in 5% CO_2_ during 1 h, fixed with 4% PFA, permeabilized with Triton X-100 0.25%, and stained with 1 μg/mL TRIT-C Phalloidin (Sigma-Aldrich) in order to observe PMN actin, and 1 μg/mL TOPRO-3 (ThermoFisher Scientific) for DNA staining, during 1 h. High resolution images were acquired using a FluoView FV1000 confocal microscope (Olympus, Tokyo, Japan) equipped with a Plapon 60x/1·42 objective lens and processed using Olympus Flow view software. First, to perform a random acquisition of images, PMN were focused on TRIT-C Phalloidin staining only, and 10 fields were observed, with at least 5 cells/field. The acquisition of each field was performed in Z-Stack mode, acquiring one frame every 1.2 μm from the upper focal plane to the lower plane. Images were analyzed using the Fiji software with the aid of the orthogonal view, in order to distinguish between internalized and adhered bacteria. Phagocytized bacterium were defined as a GFP+ particle surrounded by PMN actin, observed in both XZ and YZ planes of the orthogonal view. Values were expressed as the percentage of PMN with GFP+ bacteria internalized or adhered. The number of bacteria internalized *per* PMN was also quantified.

### Bactericidal Activity Assays

Killing of bacteria by PMNs was determined using PMNs (1 × 10^6^) combined with 1 × 10^6^ of bacteria in 24-well tissue culture plate. One hour after incubation PMN were lysed with H_2_O. The resultant solution was plated on TSA in serial dilutions. CFU were enumerated the following day, and the relative CFU was calculated with the following equation: (CFU + PMN/CFU – PMN) × 100, where CFU + PMN was the number of CFU in the presence of PMN and CFU – PMN was the number of CFU in the absence of PMN.

### Bacterial Extracts

Mechanical disruption of bacteria was performed using 0.1 mm Zirconia beads as described previously (16). Briefly, bacterial strains were cultured in 10 mL of TSB until log phase. Bacteria was pelleted by centrifugation at 9,600 g for 15 min, washed twice in PBS 1x, resuspended in 500 μL of PBS 1x with 300 mg of Zirconia beads, and then strongly vortexed during 5 min. The suspension was centrifuged at 9,600 g for 15 min and the supernatant (bacterial extract, BE) was stored.

### Protein Quantification

Protein concentration in bacteria or BE was determined using the Bradford Method (17). A standard curve using bovine serum albumin ranging from 50 to 250 μg/ml was used.

### Polysaccharide Quantification

The amount of polysaccharides was determined using the phenol-sulfuric method. Briefly, 100 μL of the BE was mixed with phenol 6% and sulfuric acid 98%, incubated during 1 h and the resultant yellow-gold color was read at 490 nm.

### Assay for Degradation of Hydrogen Peroxide (H_2_O_2_)

H_2_O_2_ degradation was evaluated over time using a DeNovix DS-11spectrophotometer (DeNovix Inc.), by means of the property of peroxide to absorb at 240 nm. H_2_O_2_ 0.036% v/v absorbance was evaluated during 240 s, with or without BE (10 μg/mL of protein). A decay control was performed by the addition of 10 Units of catalase.

### Evaluation of the Importance of Protein Content in BE

#### Heat Treatment

BE were treated for 60 min at 60°C for protein denaturalization and enzyme inactivation.

#### Trichloroacetic Acid Precipitation (TCA)

In order to precipitate proteins, TCA (15% v/v) was added to BE. The solution was incubated 30 min at −4°C and centrifuged at 9,600 g for 15 min. The supernatant was discarded, the pellet (protein fraction) was resuspended in PBS 1x and precipitated once again with pure acetone during 2 h at −4°C. After that, suspension was centrifuged and acetone was evaporated by dried air incubation. Dried protein fraction (PF) was resuspended in PBS 1x and adjusted to pH = 7.0 with NaOH 0.5 N. Final protein concentration was measured using the Bradford Method and adjusted to 100 μg/mL. TCA precipitated 97% of total proteins measured as stated in section Protein Quantification.

### Evaluation of the Importance of Polysaccharides Content in BE

#### Concanavalin a (Con A) Precipitation

Depletion of molecules containing mannose was performed by precipitation with 10 μg/mL Con A (Sigma-Aldrich) in the presence of 10 mM Ca^2^ (Merck) for 30 min at 37°C. After incubation, the solution was centrifuged at 9,600 g for 30 min. and the supernatant, free of containing mannose molecules, was reserved. Con A precipitates 75% of total carbohydrates, measured as stated in Polysaccharide Quantification.

#### Oxidation With Periodic Acid (PA)

When treated with PA, glycols are oxidized to aldehydes, losing their biological effect. In this sense, BE were oxidized with PA (Sigma-Aldrich) at a 0.1% final concentration, and stirred during 1 h. After oxidation, the solution was neutralized with NaOH 0.5 N until pH = 7.0.

### LPS Depletion and Isolation

Depletion of LPS was performed using a Polymyxin B-Agarose column (Sigma-Aldrich). The column was washed with endotoxin-free 0.1 M ammonium bicarbonate buffer (pH = 8.0), in order to remove the glycerol storage solution; then it was centrifuged at 9,600 g for 5 min and resuspended with an equal volume of BE. The polymyxin B-Agarose-BE suspension was stirred for 30 min at 37°C, and then it was centrifuged at 9,600 g for 10 min and the supernatant (BE-LPS) was reserved.

LPS was eluted from the column using 1% sodium deoxycholate (Sigma-Aldrich) and then the solution was dialyzed to eliminate the salt and resuspended in endotoxin-free water.

### Flow Cytometry Studies

5 × 10^5^ PMN were incubated with a specific mouse anti-human CD11b antibody conjugated with phycoerythrin (PE) (Dako, Santa Clara, CA, USA). Debris was excluded by FSC-SSC, and the increase on FSC or CD11b expression was analyzed within the gated-viable PMN. Mean fluorescence intensity of the CD11b was determined on 50.000 events.

### Measurement of Fluctuations in Intracellular Ca^2+^

Changes in intracellular Ca^2+^ were monitored using Fluo-3AM (Sigma Aldrich), as previously described (18). Briefly, neutrophils suspended at a concentration of 5 × 10^6^ cells/ml in RPMI 1640 were incubated with 4 mM Fluo-3AM for 20 min at 30°C. Then loaded cells were washed twice and suspended at 5 × 10^6^ cells/ml in RPMI 1640 supplemented with 1% FCS. Aliquots of 15 μl of this cell suspension were added to 300 μl of RPMI 1640 medium containing 1% FCS and warmed at 37°C. The samples were immediately loaded onto the flow cytometer, and the basal fluorescence (FL-1) was recorded during 15 s. Then cells were stimulated with fMLP (10^−7^ M), and the fluorescence was recorded during an additional 150 s. Acquisition of samples was performed at 37°C. Fluctuations in cytoplasmatic free calcium concentrations were recognized as alterations in Fluo-3AM fluorescence intensity over time.

### Reactive Oxygen Species (ROS) Generation

To determine the production of ROS by flow cytometry DHR-123, a derivative of rhodamine 123, was used following the protocol described by Leech et al. (19). Briefly, isolated PMN (5 × 10^5^) were incubated 15 min at 37°C with 1 μM DHR-123. Subsequently, the cells were incubated with or without the stimuli for 30 min at 37°C 5% CO_2_ in a humidified atmosphere. Immediately after, the green fluorescence was determined.

### Neutrophil Extracellular Traps (NETs) Formation

PMN (5 × 10^5^) were seeded gently onto glass coverslips coated with 0.001% poly-L-lysine in a 24-well plate in triplicate, allowed to settle, and incubated in the presence of bacteria (bacteria:PMN ratio 20:1). Cells were incubated for 3 h at 37°C 5% CO_2_. After the incubation period, samples were gently fixed with 4% PFA, then washed with PBS 1x, and stained for DNA with propidium iodide (Vector Laboratories) and elastase using a specific antibody anti-PMN elastase (Merk Millipore, Darmstadt, Germany). Images for NETs evaluation were acquired using a FluoView FV1000 confocal microscope (Olympus, Tokyo, Japan) equipped with a Plapon 60x/1·42 objective lens and processed using Olympus. At least 10 different fields were observed in each triplicate (× 200). NETs areas were determined as previously reported (20) in at least five pictures obtained in × 200 using the wand tool of the FIJI software (21). The scale for the measurement was obtained from the data given in the confocal microscope image.

### Chemotaxis

Chemotaxis was quantified using a modification of the Boyden chamber technique (22). A cell suspension (50 μl) containing 2 × 10^6^ cells/ml in RPMI with 2% FCS, was placed in the top wells of a 48-well micro-chemotaxis chamber. A PVP-free polycarbonate membrane (3 μm pore size; Neuro Probe Inc. Gaithersburg MD, USA) separated the cells from lower wells containing either RPMI or the stimulus. The chamber was incubated for 30 min at 37°C in a 5% CO_2_ humidified atmosphere. After incubation, the filter was stained with TINCION-15 (Biopur SRL, Rosario, Argentina), and the number of PMN on the undersurface of the filter was counted in a five random high-power fields (HPF) × 400 for each of triplicate filters.

### Statistical Analysis

Results were expressed as the mean ± SEM. Statistical analysis of the data was performed using the analysis of variance (ANOVA), applying Tukey's post-test. *P* < 0.05 were considered significant.

## Results

### Kpn KPC ST258 Triggers a Poor Respiratory Burst in PMN

**Figure 1:**
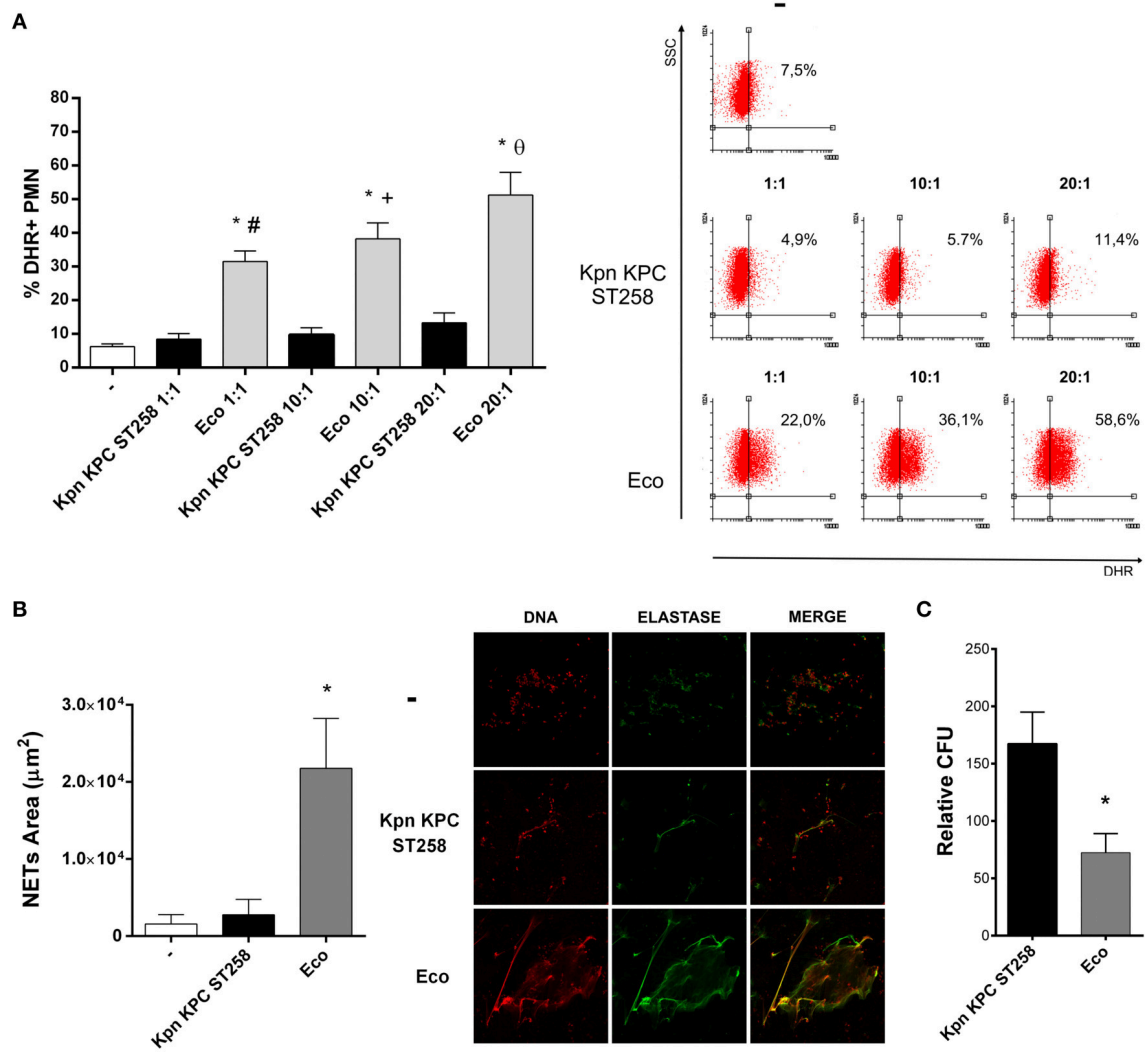
Kpn KPC ST258 is a poor inducer of ROS generation. Kpn KPC ST258 and Eco were incubated with isolated human PMN in different Bacteria:PMN ratios for 30 min. ROS was measured by flow cytometry using DHR. **(A)** Percentage (%) of DHR + PMN. Right panel: Representative dot-plots showing the SSC vs. DHR profiles. *n* = 10; ^*^p < 0.05 vs. untreated (-); # *p* < 0.05 vs. Kpn KPC ST258 1:1, +*p* < 0.05 vs. Kpn KPC ST258 10:1; θ *p* < 0.05 vs. Kpn KPC ST258 20:1. **(B)** Isolated PMN were incubated with Kpn KPC ST258 and Eco in a Bacteria:PMN ratio of 20:1 for 3 h. NETs were stained for DNA and elastase and were visualized by confocal microscopy. NETs area (μm^2^) was measured using the FIJI software as described in material and methods. *n* = 10. ^*^p < 0.05 vs. untreated (-) and vs. Kpn KPC ST258. Right panel: Representative microphotographs of confocal images showing PMN from the untreated (-), Kpn KPC ST258, and Eco groups stained for DNA or elastase visualization and the merge of the images (x 200). **(C)** Kpn KPC ST258 or Eco were incubated for 1 h with PMN and the remaining viable colony forming units (CFU) were evaluated using TSA plates. Relative CFU was calculated as indicated in material and methods. *n* = 9. ^*^p < 0.05 vs. Kpn KPC ST258. In all cases, results are expressed as the mean ± SEM.

Considering the clinical importance of the hyper-epidemic clone of Kpn producer of carbapenemase (KPC) that belongs to the ST258 (Kpn KPC ST258), we focused our study on this bacterial clone. We first investigated the respiratory burst, which plays an important role in innate immunity against invading microorganisms using different bacteria:PMN ratios. The fluorogenic dye, dihydrorhodamine 123 (DHR), was used for detection of reactive oxygen species (ROS). As shown in Figure 1A, ROS generation was strongly induced after 30 min when PMN were challenged with Eco. However, Kpn KPC ST258 was not able to induce a respiratory burst in PMN in any of the ratios assayed. The possibility that Kpn KPC ST258 was a slower inducer of ROS was ruled out as the percentages of ROS-producing PMN at 1 and 2 h were 8.8 and 10.8%, respectively, and were not different from the percentage observed at 30 min (data not shown).

The viability of PMN was measured in parallel by flow cytometry using propidium iodide, and none of the conditions assayed induced cell death (Supplementary Material 2). We decided to use a bacteria:PMN ratio of 20:1 to perform all the experiments that followed.

We next wanted to address the importance of carrying KPC in the lack of ROS induction by Kpn. We performed the assay using other Kpn strains: one ATCC, not ST258, three other clinical isolates of Kpn ST258 but negative for KPC, and three other clinical isolates of Kpn ST258 positive for KPC. We found that all ST258 clinical isolated of Kpn were unable to induce a respiratory burst in PMN independently of the presence of KPC (Supplementary Material 3). Moreover, the ATCC strain was also a poor inducer of ROS in PMN.

As the production of ROS has been associated with the capacity of PMN to form NETs (23), we also evaluated NETosis triggered by Kpn KPC ST258. Figure 1B shows that Kpn KPC ST258 was also a poor inducer of NETosis after 3 h of incubation, whereas Eco induced a significant amount of NETs.

Next, to evaluate whether the poor induction of ROS and NETs by Kpn results in increased bacterial survival, PMN were incubated with Kpn KPC ST258 or Eco for 1 h. Then, cells were lysed and total colony formation units (CFU) were determined on agar plates. As shown in Figure 1C, the CFU count for Kpn KPC ST258 was higher compared to Eco.

Altogether, these results indicate that Kpn KPC ST258 is a poor inducer of important PMN bactericidal mechanisms, and this is associated with higher bacterial survival.

### PMN Are Able to Sense Kpn KPC ST258

Taking into account the results found in the previous section, we next asked if Kpn KPC ST258 could be sense by PMN. If PMN recognizes Kpn, the lack of response would not be due to the lack of interaction/ingestion, and could be associated with some type of active modulatory mechanism of PMN functionality fulfilled by the bacterium.

**Figure 2:**
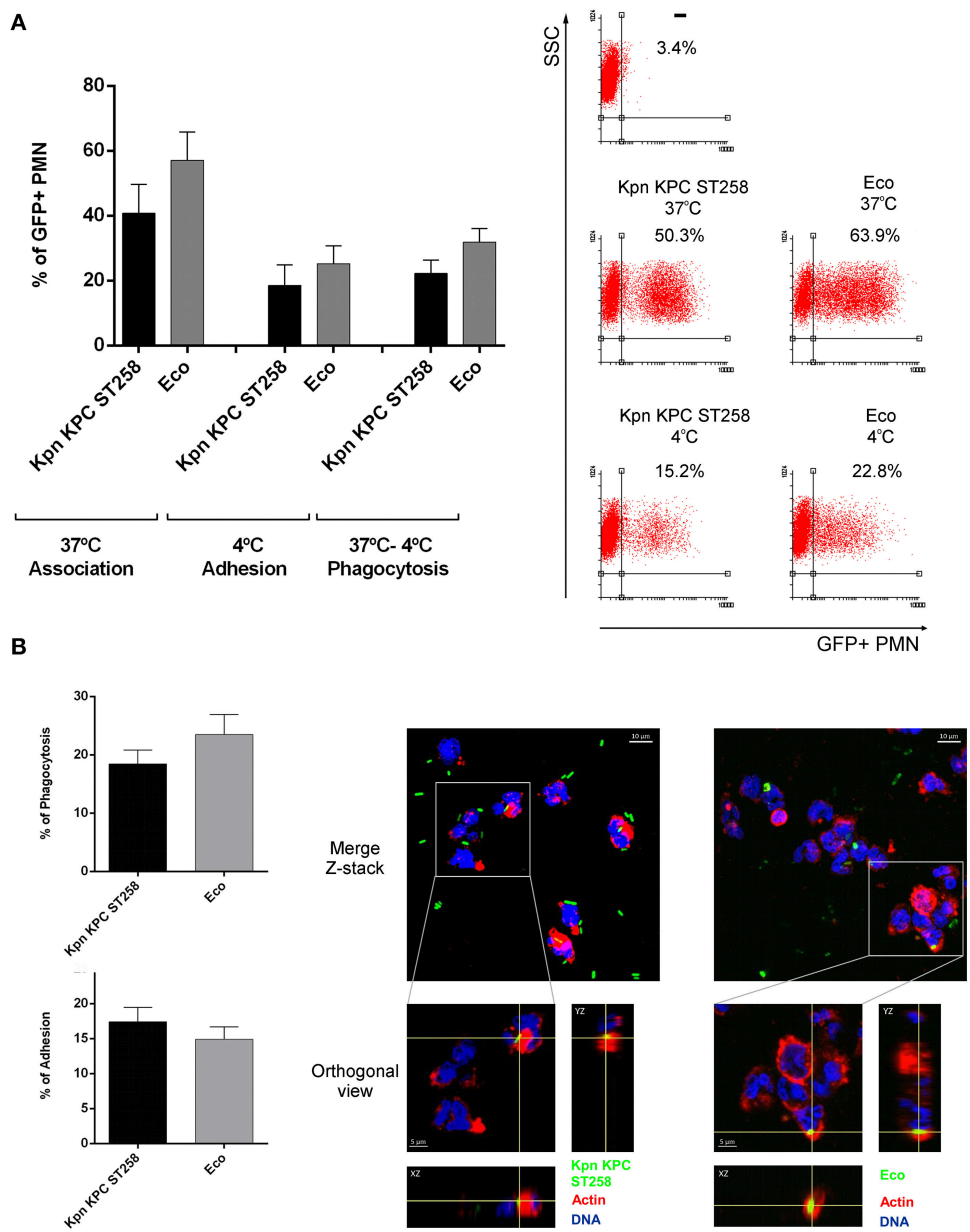
Kpn KPC ST258 and Eco are similarly phagocytized by PMN. Percentage of phagocytosis and bacterial attachment were evaluated by both flow cytometry **(A)** and confocal microscopy **(B)** techniques. GFP-Kpn KPC ST258 and GFP-Eco were incubated with isolated human PMN as described in material and methods. **(A)** Percentage of association and adhesion of GFP-bacteria to PMN were measured at 37°C and 4°C, respectively. Percentage of PMN that have phagocytized GFP-Bacteria was calculated by subtraction (37°C–4°C). *n* = 8. Right panel: Representative Dot-Plots. **(B)** Percentage of PMN with phagocytized or adhered GFP-Bacteria evaluated by confocal microscopy. *n* = 5. Right panel: representative Z-Stack merged microphotographs and orthogonal view, showing PMN with phagocytized GFP-Bacteria. Actin was stained with TRIT-C phalloidin (red) and DNA with TOPRO-3 (blue). Images were analyzed using Fiji software as described in materials and methods. In all cases results were expressed as the mean ± SEM.

Therefore, in order to investigate bacteria-PMN interaction, we used GFP-recombinant Kpn KPC ST258 and GFP-Eco and analyze the percentage of phagocytosis after 1 h of incubation by flow cytometry and confocal microscopy (Figures 2A,B). Using both techniques, attached and internalized bacteria could be distinguished. Similar results were obtained using the two different techniques and the percentage of PMN that have attached or phagocytized GFP-Kpn KPC ST258 or GFP-Eco was similar. Moreover, using the confocal images we quantified the number of bacteria internalized *per* PMN, and again the results obtained were similar for Kpn KPC ST258 and Eco (No. bacteria internalized *per* PMN: Kpn KPC ST258 = 1.17 ± 0.18, Eco = 1.20 ± 0.04, *n* = 5).

**Figure 3:**
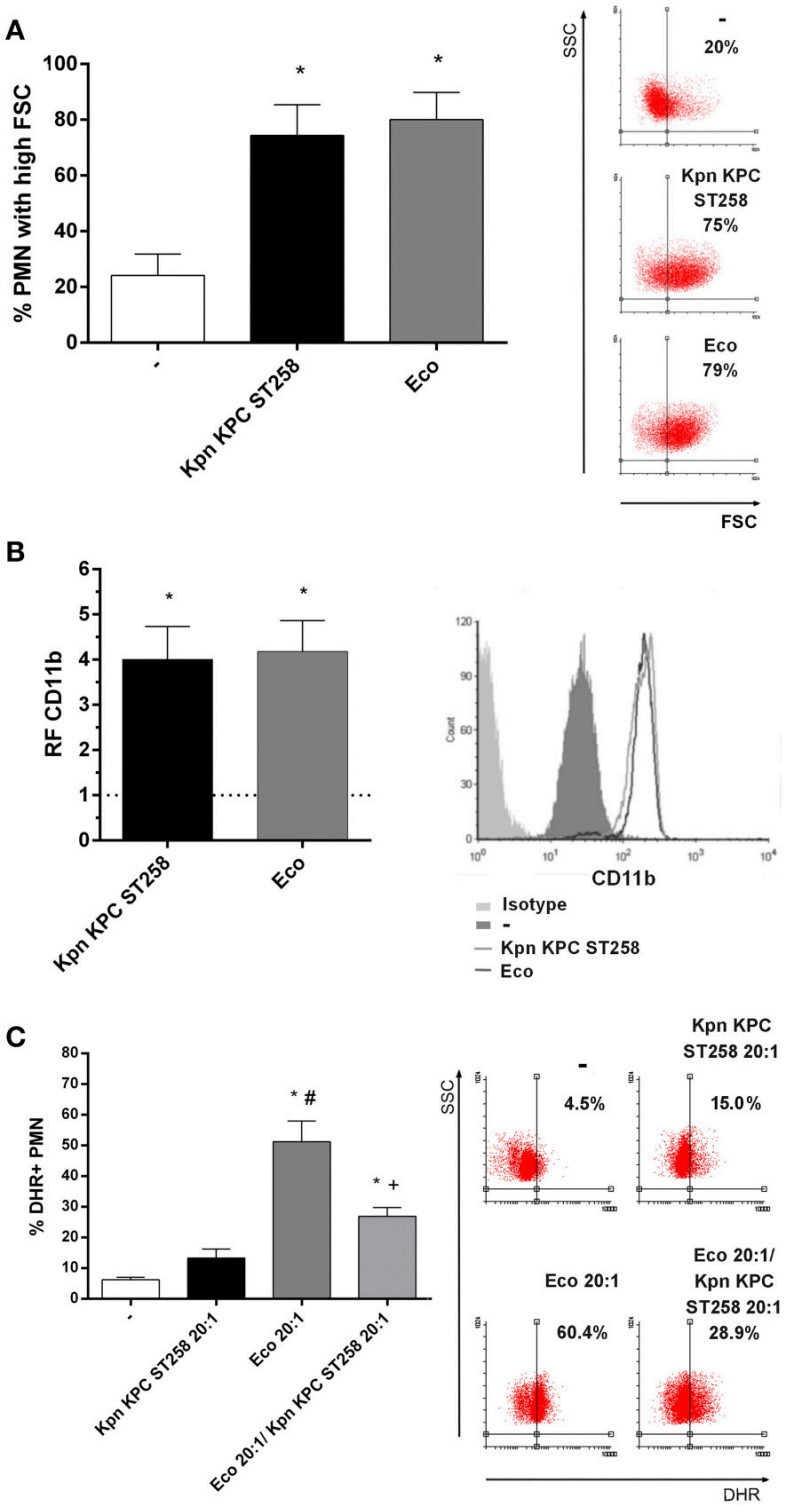
Kpn KPC ST258 is recognized by PMN. Kpn KPC ST258 and Eco were incubated with isolated human PMN in a Bacteria:PMN ratio of 20:1 for 30 min. **(A)** The % of PMN that increased their Forward Size Scatter (FSC) after stimulation with Kpn KPC ST258 or Eco, or left untreated (-) was determined by flow cytometry. Results were expressed as the mean ± SEM, *n* = 8. ^*^p < 0.05 vs. (-). Right panel: representative dot-plots showing SSC vs. FSC profiles of PMN with no treatment (-), Kpn KPC ST258 or Eco. **(B)** Expression of CD11b. The MFI of the adhesion marker CD11b was determined by flow cytometry. The horizontal dashed-line represents the CD11b expression of the control group. Results were expressed as the mean ± SEM of the relative fluorescence (RF) of the MFI of the different treatments with respect to untreated PMN. *n* = 5. ^*^p < 0.05 vs. (-). Right panel: Representative histogram of the expression of CD11b in the different experimental groups. **(C)** % of DHR+ PMN of Kpn KPC ST258-Eco co-cultures. Results were expressed as the mean ± SEM. *n* = 5. ^*^p < 0.05 vs. untreated (-); # *p* < 0.05 vs. Kpn KPC ST258 20:1, +*p* < 0.05 vs. Eco 20:1. Right panel: representative dot-plots showing SSC vs. DHR profiles.

To determine whether this bacteria-PMN interaction leads to PMN activation, we measured the increase in their FSC, a rapid and sensitive parameter related to initial sensing of inflammatory/infectious stimuli. Kpn KPC ST258 was equally capable of inducing an FSC increase in PMN compared to Eco (Figure 3A). Moreover, initial steps in PMN activation involve the mobilization of intracellular granules, resulting in the up-regulation in the plasma membrane of molecules that were present in the membrane of intracellular granules. This is the case of CD11b that was measured on the surface of PMN after incubation with the different bacterial strains. As depicted in Figure 3B, the up-regulation of CD11b in PMN was similar for Kpn KPC ST258 and Eco. Altogether, these results indicate that Kpn KPC ST258 was interacting with PMN, and although PMN was similarly sensing Kpn KPC ST258 and Eco, for Kpn, this sensing caused dual effects on PMN, modulating some functions positively, but negatively regulating others.

Moreover, in order to investigate whether Kpn KPC ST258 was able to interfere with ROS induced by other bacteria, we co-cultured PMN with Kpn KPC ST258 and Eco and found that Kpn KPC ST258 was able to decrease the respiratory burst triggered by Eco (Figure 3C). Again, the diminished ROS generation was not due to PMN death caused by bacterial mixture (Supplementary Material 2). This result indicates that Kpn KPC ST258 is delivering a negative signal in PMN that also affects the response to other stimuli.

### A Bacterial Cell Wall Component of Kpn KPC ST258 Inhibits ROS Generation Triggered by fMLP

**Figure 4:**
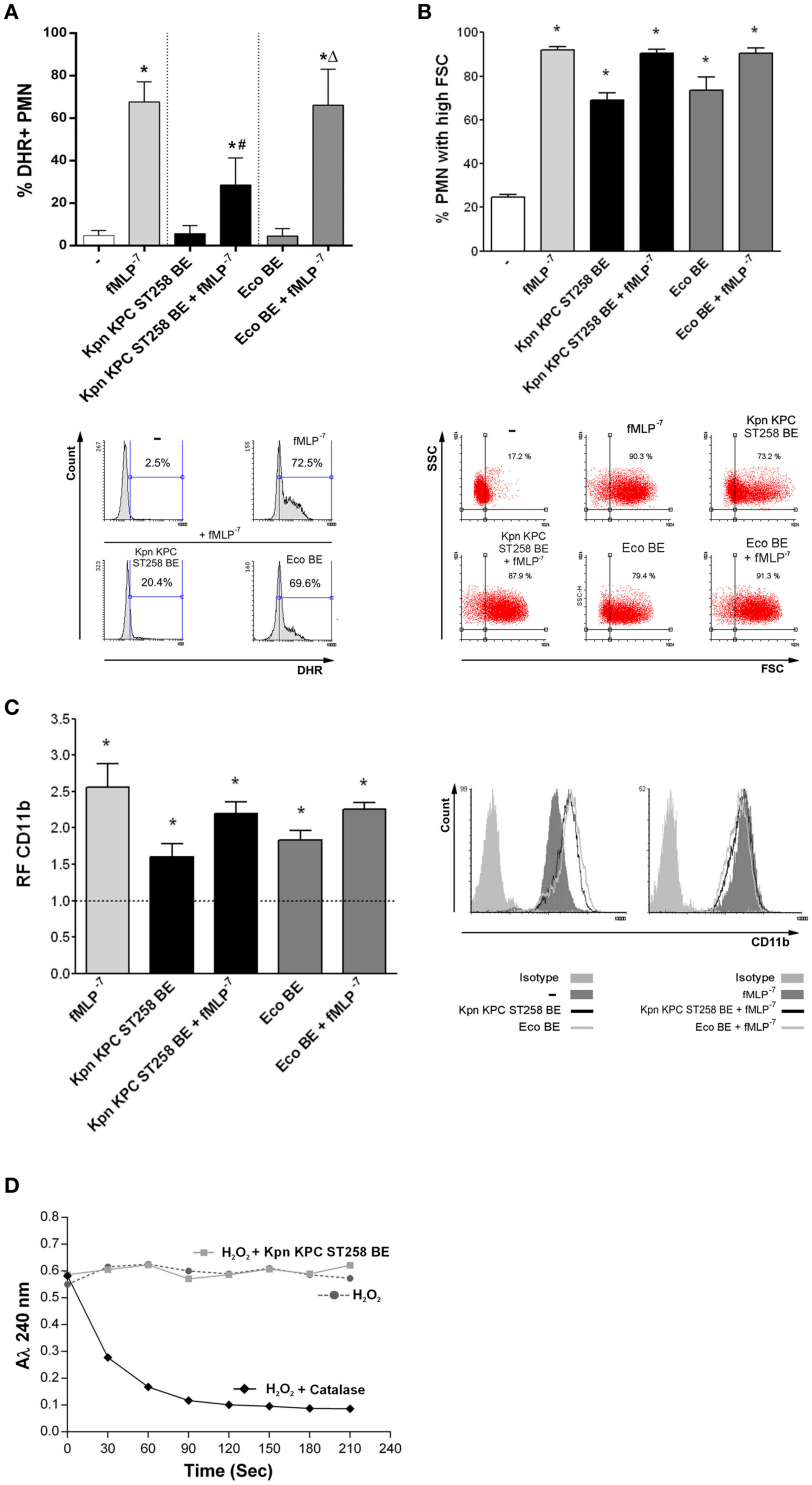
A bacterial cell wall component of Kpn KPC ST258 inhibits ROS generation. Bacterial extracts (BE) from Kpn KPC ST258 or Eco were obtained and PMN were incubated for 30 min with these BE (10 μg/mL of total protein) in the presence or absence of fMLP (10^−7^ M). **(A)** ROS generation. The percentage of DHR+ PMN was determined by flow cytometry. *n* = 10. Results were expressed as the mean ± SEM. ^*^p < 0.05 vs. untreated (-); # *p* < 0.05 vs. fMLP^−7^; Δ*p* < 0.05 vs. Kpn KPC ST258 BE + fMLP^−7^. Lower panel: Representative histograms of SSC vs. DHR. **(B)** FSC increase. The percentage of PMN that increased their FSC was determined by flow cytometry. *n* = 10. Results were expressed as the mean ± SEM. ^*^p < 0.05 vs. (-). Lower panel: Representative dot-plots of SSC vs. FSC profiles. **(C)** CD11b expression. The MFI of the adhesion marker CD11b was determined by flow cytometry and expressed as the relative fluorescence (RF) of the MFI of the different treatments with respect to untreated PMN. *n* = 10. Results were expressed as the mean ± SEM; ^*^p < 0.05 vs. (-). Right panel: Representative histograms of CD11b expression. **(D)** H_2_O_2_ degradation over time. The absorbance at 240 nm was recorded for H_2_O_2_ (0.036% v/v) in the presence of catalase (10 U) or Kpn KPC ST258 BE (10 μg/mL of total protein). *n* = 3.

In order to study which component was involved in the regulation of ROS by Kpn KPC ST258, we performed bacterial extracts (BE) enriched in bacterial cell wall components and used these BE in a ROS production assay, triggering ROS with fMLP, a well-known ROS inducer. The use of strong positive inducer is critical to evidence an active inhibitory mechanism. Moreover, we also used Eco BE to determine whether ROS modulation was exclusive for Kpn. The concentration of BE used was approximately equivalent to the amount of protein found in 1 × 10^7^ CFU, corresponding to a bacteria:PMN ratio of 20:1. Even though none of the BE by themselves were able to trigger ROS in PMN, when we evaluated the modulation of ROS generation induced with fMLP by BE (Figure 4A), we observed a statistically significant reduction in the % of ROS-producing PMN by Kpn KPC ST258 BE, whereas Eco BE did not affect the percentage of ROS-producing PMN.

As the main components of BE are proteins and polysaccharides, we quantified both types of molecules in BE from Kpn KPC ST258 and Eco and performed the ROS generation measurements normalizing Kpn KPC ST258 and Eco BE according to their protein or polysaccharide content. The inhibition of ROS generation caused by Kpn KPC ST258 BE was similar when BE concentrations were normalized according to their protein or polysaccharide content (Supplementary Material 4).

In contrast to the inhibition observed for ROS generation, and similarly to what was observed with the whole bacteria, BE from Kpn KPC ST258 and Eco were equally able to increase the FSC of PMN and their CD11b expression (Figures 4B,C). When incubated together with fMLP, none of the BE negatively modulated the FSC and CD11b expression increases induced by fMLP.

Considering the above result, the first issue we wanted to exclude was that BE from Kpn KPC ST258 may contain components that were able to scavenge or directly degrade hydrogen peroxide (H_2_O_2_), the product detected in the ROS generation assay used in this study. For this purpose, we measured H_2_O_2_ degradation over time, by means of the property of H_2_O_2_ to absorb at 240 nm. As shown in Figure 4D, the addition of catalase, used as a positive control, caused a rapid decrease in the absorbance of H_2_O_2_, whereas Kpn KPC ST258 BE did not affect H_2_O_2_ levels over time. This result indicates that the component responsible for fMLP-induced ROS inhibition is not scavenging H_2_O_2_.

### Mechanisms Involved in Kpn KPC ST258 BE-Mediated Inhibition of fMLP-Induced ROS

**Figure 5:**
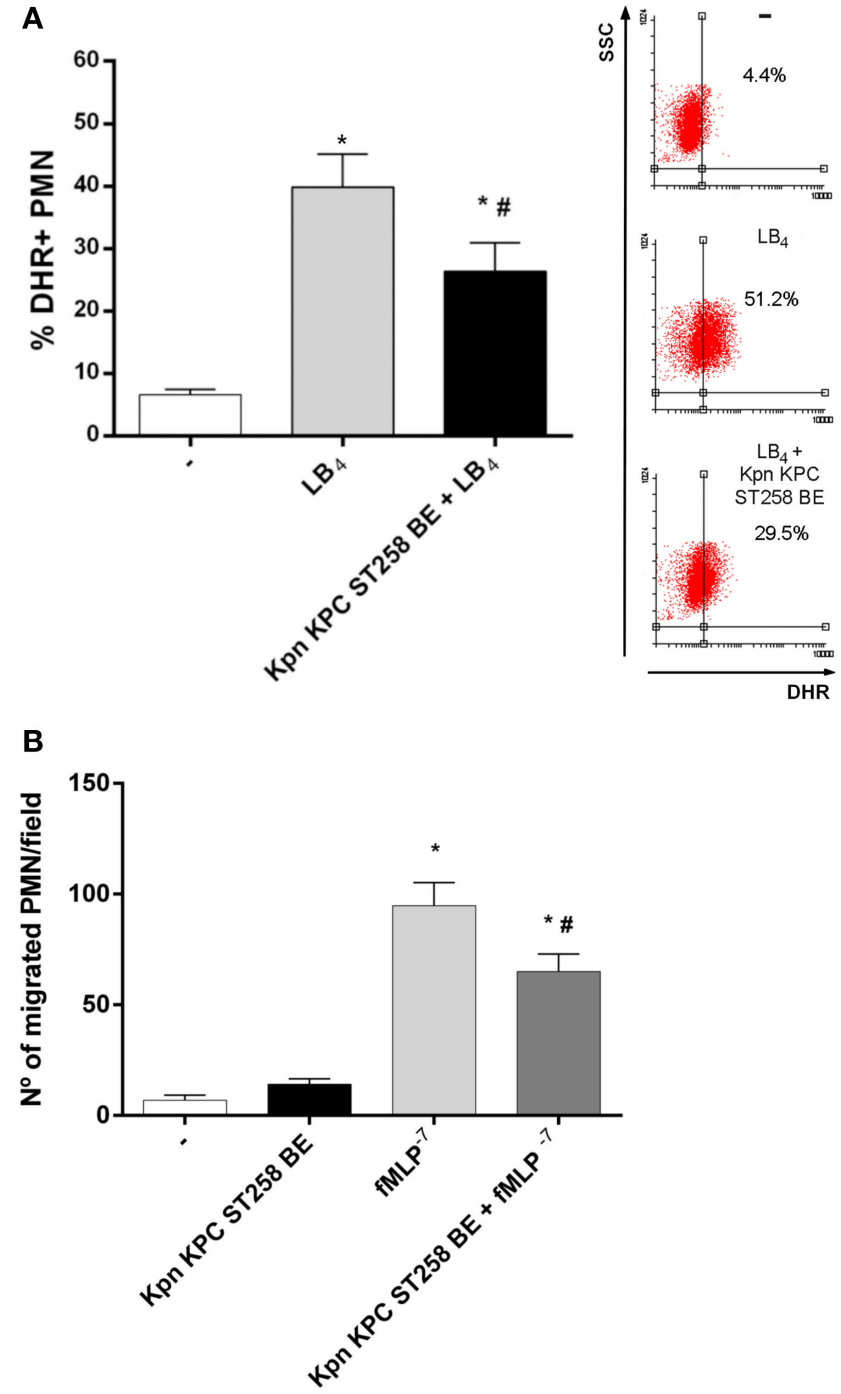
BE from Kpn KPC ST258 inhibits ROS mediated by leukotriene B_4_ (LB_4_), and also negatively modulates other functions mediated by fMLP. **(A)** ROS was triggered by LB_4_ (0.1 μg/mL) in the presence or absence of Kpn KPC ST258 BE and the % of DHR+ PMN was measured by flow cytometry. Results were expressed as the mean ± SEM. Right panel: Representative dot-plot showing SSC vs. DHR profiles. *n* = 7; ^*^p < 0.05 vs. untreated (-); # *p* < 0.05 vs. LB_4_. **(B)** Chemotactic response using a Boyden chamber. The number of PMN that have migrated toward fMLP (10^−7^ M) through a 3 μm-pore membrane in the presence or absence of Kpn KPC ST258 BE is shown. Results were expressed as the mean ± SEM, *n* = 6; ^*^p < 0.05 vs. (-); # *p* < 0.05 vs. fMLP^−7^.

PMN's fMLP receptor belongs to the family of G-protein-coupled seven-transmembrane receptors (24). We wanted to address if Kpn KPC ST258 BE may be also affecting ROS generation triggered in response to other stimuli that signal by a different receptor of the same family of seven-transmembrane receptors. As shown in Figure 5A, leukotriene B_4_ (LB_4_) was able to induce ROS, and the presence of Kpn KPC ST258 BE partially abolished this induction. Therefore, the inhibition of ROS by BE is not specific for fMLP, and it may affect a common signaling pathway triggered by other G-protein-coupled seven-transmembrane receptors.

Then, we asked whether Kpn KPC ST258 could also modulate other PMN functions triggered by fMLP. We evaluated chemotaxis toward fMLP using a Boyden chamber in the presence or absence of Kpn KPC ST258 BE and found that, similarly to what was observed in ROS generation, the presence of Kpn KPC ST258 BE partially decreased the number of PMN that migrate toward fMLP (Figure 5B).

**Figure 6:**
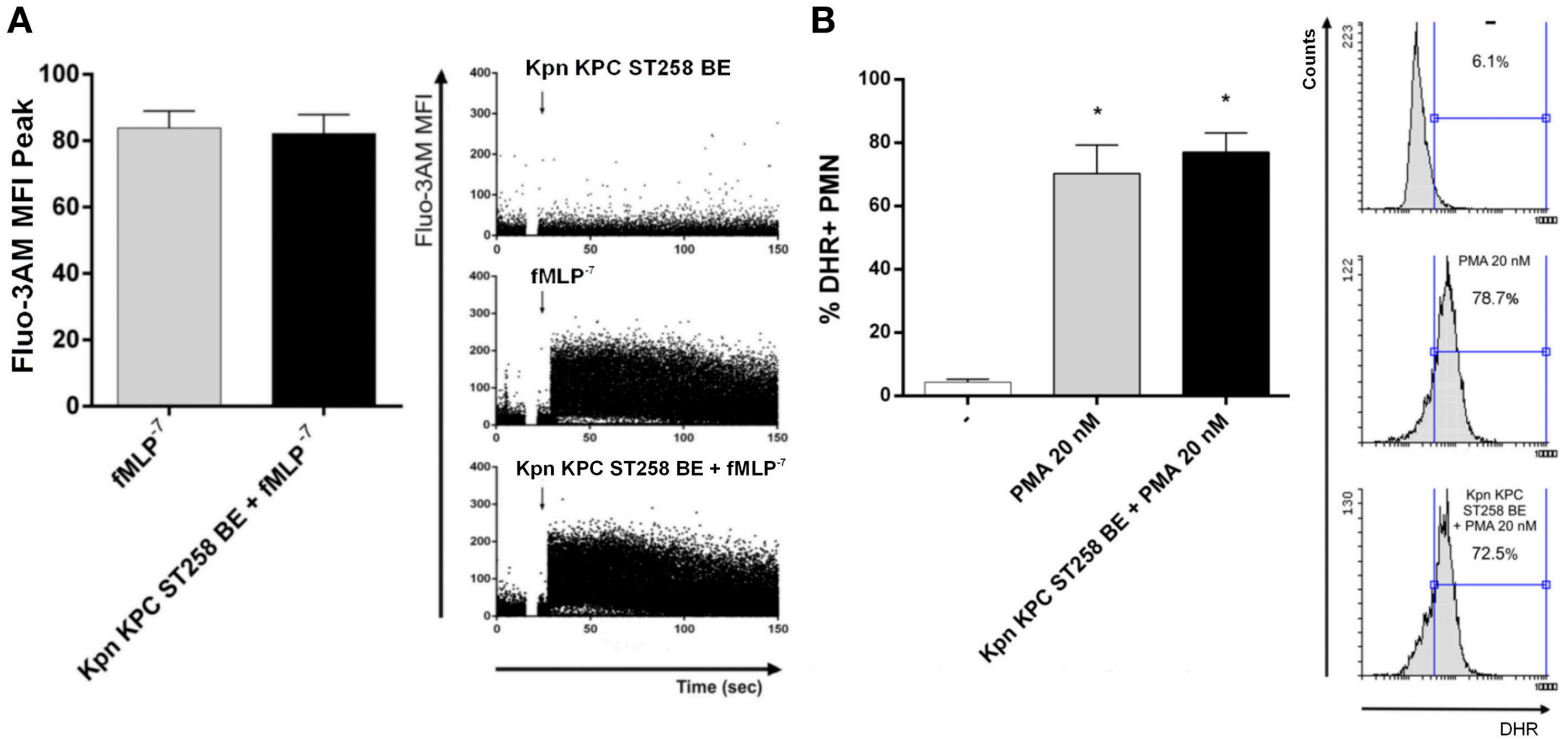
**(A)** Intracellular Ca^2+^ mobilization. PMN were incubated with Fluo 3-AM and Ca^2+^ mobilization was triggered by fMLP in the presence or absence of Kpn KPC ST258 BE and the mean ± SEM of the peak MFI of Fluo 3-AM is shown. *n* = 6. Right panel: Representative dot-plots profiles of one representative experiment out of three showing the Fluo 3-AM fluorescence over time. **(B)** ROS triggered by PMA in the presence or absence of KPC ST258 BE. Results were expressed as the mean ± SEM of the % of DHR+ PMN. *n* = 7. ^*^p < 0.05 vs. (-). Right panel: Representative histograms showing SSC vs. DHR profiles.

We next wanted to study the mechanism involved in the inhibition of fMLP-induced ROS production mediated by Kpn KPC ST258. One of the first steps in fMLP signaling is the mobilization of Calcium (Ca^2+^) from intracellular stores (24). In order to evaluate whether the inhibitory component present in Kpn KPC ST258 BE was affecting Ca^2+^ mobilization, we used a Fluo 3-AM probe and evaluated intracellular variations in Ca^2+^ levels by flow cytometry measuring the increase in the fluorescence of this reactive over time, in the presence or absence of Kpn KPC ST258 BE, using fMLP as the stimulus. As depicted in Figure 6A, the intracellular Ca^2+^ mobilization induced by fMLP was not affected by the presence of Kpn KPC ST258 BE. These results indicate that Kpn KPC ST258 BE is not inhibiting fMLP-induced ROS by affecting Ca^2+^ mobilization.

Another possibility was that Kpn KPC ST258 BE could be interfering with the final steps necessary for ROS induction, that is, NADPH oxidase assembly. PMA is often used to bypass cell surface receptors and induce a more direct activation of the NADPH oxidase via a direct protein kinase C-mediated phosphorylation of p47^*phox*^ (25). Therefore, we evaluated ROS production using PMA as a stimulus in the presence or absence of Kpn KPC ST258 BE (Figure 6B). We found that PMA induced a strong respiratory burst in PMN, and the presence of Kpn KPC ST258 BE was not able to modulate it, indicating that the component/s of Kpn KPC ST258 BE that are inhibiting ROS production are not acting at the NADPH oxidase assembly level.

### The Inhibitory Component of Kpn KPC ST258 BE Is a Polysaccharide

**Figure 7:**
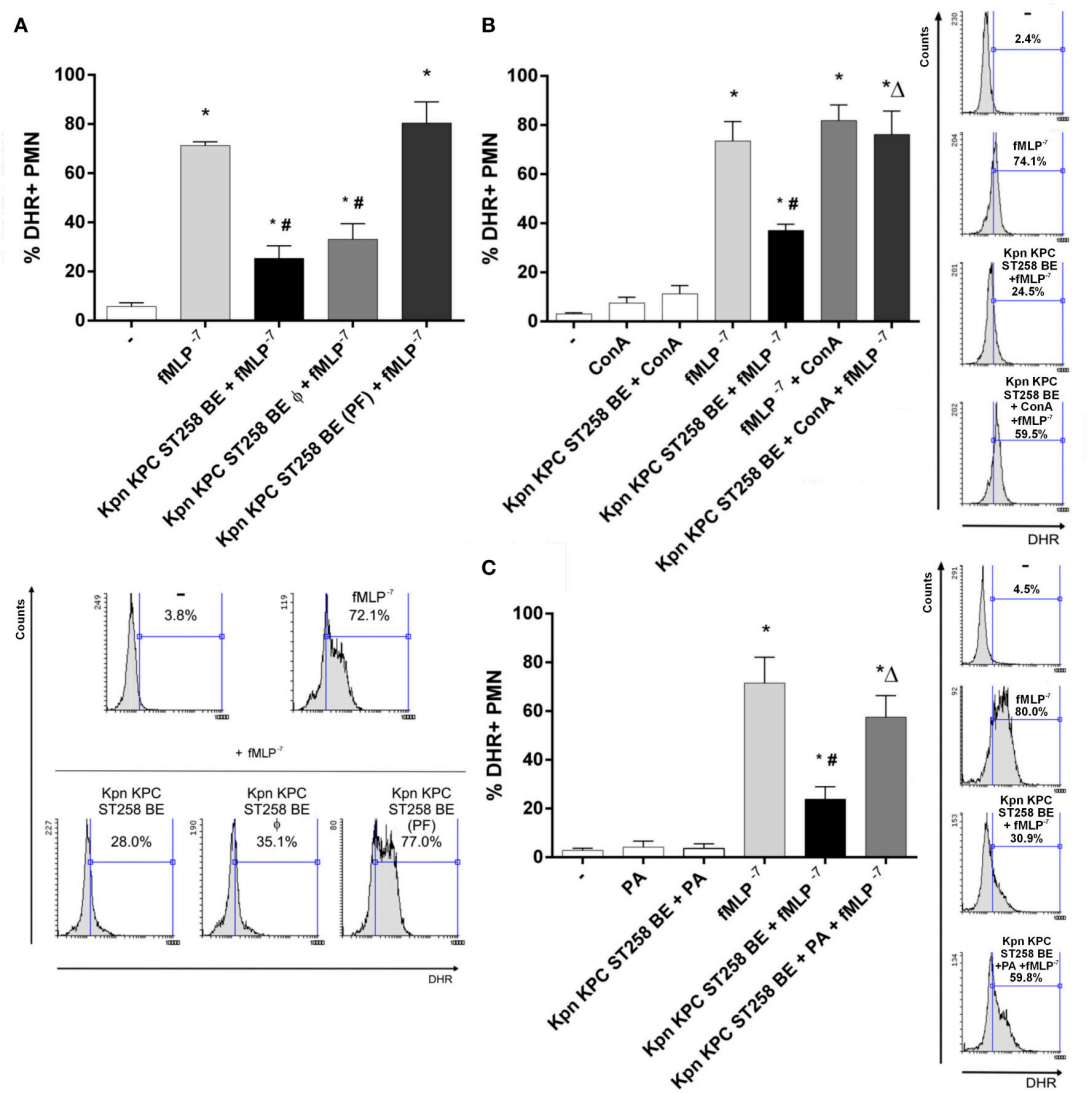
Unraveling the nature of the inhibitory component in KPC ST258 BE. **(A)** fMLP-triggered ROS was measured in PMN in the presence of heat-treated (Φ) KPC ST258 BE or in protein-precipitated fractions (PF). Results of the % of DHR+ PMN were expressed as the mean ± SEM. Lower panel: representative histogram showing SSC vs. DHR. *n* = 7. ^*^p < 0.05 vs. untreated (-); # *p* < 0.05 vs. fMLP^−7^. **(B)** Mannose-containing molecules in KPC ST258 BE were depleted by precipitation with Concanavalin A (Con A) and this depleted fraction was assayed for its inhibitory potential in fMLP-induced ROS. The % of DHR+ PMN is shown and results were expressed as the mean ± SEM, *n* = 7. ^*^p < 0.05 vs. untreated (-); # *p* < 0.05 vs. fMLP^−7^; Δ*p* < 0.05 vs. Kpn KPC ST258 BE + fMLP^−7^. Right panel: Representative histograms showing SSC vs. DHR. **(C)** Oxidation of polysaccharides in KPC ST258 BE was performed by periodic acid (PA) treatment and ROS was triggered by fMLP in the presence of these oxidized-BE. Results (% of DHR+ PMN) were expressed as the mean ± SEM. *n* = 5. ^*^p < 0.05 vs. untreated (-); # *p* < 0.05 vs. fMLP^−7^; Δ*p* < 0.05 vs. Kpn KPC ST258 BE + fMLP^−7^. Right panel: Representative histograms showing SSC vs. DHR.

In order to investigate the nature of the component that mediates the inhibition of fMLP-induced ROS generation, we first evaluated the possible contribution of proteins in this phenomenon. We performed a heat treatment of Kpn KPC ST258 BE (60 min at 60°C) to denature proteins and abolish any enzymatic activity. As Figure 7A shows, the ability of Kpn KPC ST258 BE to inhibit ROS generation by fMLP was not modified by heat treatment. Moreover, to exclude the possible participation of a structural component of proteins, independently of their enzymatic activity, protein precipitation was performed, and this precipitated fraction (PF) was used to evaluate its direct inhibitory potential. We found that precipitated proteins from BE were not able to inhibit fMLP-induced ROS production.

Polysaccharides are the other major component of bacterial cell walls. Taking into account that mannose is a usual polysaccharide residue found in bacterial walls, and considering that concanavalin A (Con A) binds mannose residues and is able to precipitate mannose-containing molecules (26), we incubate Kpn KPC ST258 BE with Con A, and then centrifuged this Con A-treated Kpn KPC ST258 BE in order to deplete mannose-containing molecules from these BE. As depicted in Figure 7B, Con A treatment abolished the ability of Kpn KPC ST258 BE to inhibit fMLP-induced ROS generation. Moreover, polysaccharides can be oxidized by periodic acid (PA) losing their native conformation (27). Treatment of Kpn KPC ST258 BE with PA also abolished Kpn KPC ST258 BE's ability to inhibit fMLP-induced ROS generation (Figure 7C).

Altogether, these results indicate that the inhibitory ability of Kpn KPC ST258 BE is not dependent on a protein but is mediated by a mannose-containing molecule. More specifically, we could identify the direct participation of a polysaccharide in the inhibitory phenomenon.

### The Polysaccharide Part of LPS of Kpn KPC ST258 Inhibits fMLP-Induced ROS

**Figure 8:**
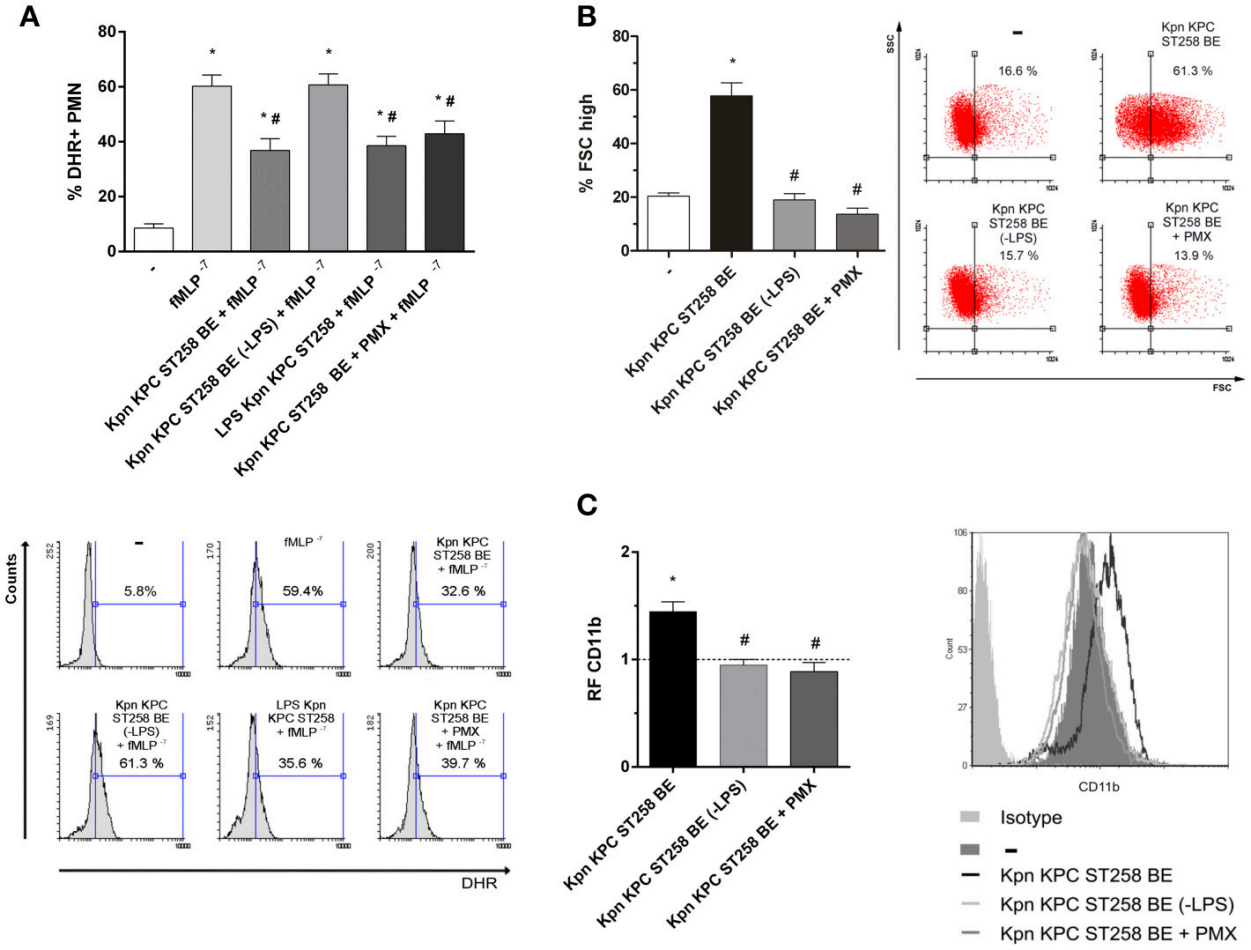
Depletion of LPS in KPC ST258 BE reversed the inhibition of ROS. **(A)** LPS from KPC ST258 BE was depleted using a polymyxin b (PMX)-agarose columns. LPS-depleted fractions (-LPS) were used to assay fMLP-induced ROS generation. Additionally, incubation of KPC ST258 BE with soluble PMX was performed and the mixture KPC ST258 BE+PMX was also used. Results were expressed as the mean ± SEM of the % of DHR+ PMN. *n* = 10. ^*^p < 0.05 vs. untreated (-); # *p* < 0.05 vs. fMLP^−7^. Lower panel: Representative histograms showing SSC vs. DHR. **(B)** The percentage of PMN that increased their FSC (% FSC high) was measured by flow cytometry in KPC ST258 BE depleted from LPS (-LPS) and in KPC ST258 BE+PMX. Results were expressed as the mean ± SEM. *n* = 10. ^*^p < 0.05 vs. untreated (-); # *p* < 0.05 vs. Kpn KPC ST258 BE. Right panel: Representative dot-plots showing SSC vs. FSC profiles. **(C)** CD11b expression was measured in the different groups by flow cytometry and results were expressed as the relative fluorescence (RF) of the MFI with respect to untreated PMN. The horizontal dashed-line represents the CD11b expression of the control group. Results were expressed as the mean ± SEM. *n* = 6. ^*^p < 0.05 vs. untreated (-); # *p* < 0.05 vs. Kpn KPC ST258 BE. Right panel: Representative histogram showing MFI expression of CD11b for the different experimental groups.

Lipopolysaccharide (LPS) is a major component of Gram-negative bacteria membranes. LPS is localized in the outer layer of the membrane and is, in non-capsulated strains as Kpn KPC ST258, exposed on the cell surface. LPS is comprised of a hydrophilic polysaccharide and a hydrophobic component known as lipid A. The antibiotic Polymyxin B (PMX) interacts with the lipid A of LPS and it can be used, when conjugated to agarose beads, to deplete the entire LPS molecule from solutions. When Kpn KPC ST258 BE were depleted using this PMX column, and the depleted fraction (-LPS) was used in ROS production triggered by fMLP, we found that depletion of LPS completely abolished BE's ability to inhibit ROS (Figure 8A). In accordance, when LPS was recovered from the column by elution with sodium deoxycholate, dialyzed and this purified LPS was evaluated for its inhibitory properties, we observed a direct inhibition of LPS on fMLP-induced ROS. Moreover, we used the PMX ability to neutralize lipid A-mediating effects to confirm the involvement of the polysaccharide part of LPS in the inhibition of ROS generation. For this purpose, soluble PMX was added to BE and the mixture Kpn KPC ST258 BE+PMX was added to PMN for evaluation. In this case, the addition of PMX in BE did not reverse the inhibitory effect mediated by Kpn KPC ST258 BE, ruling out any role of lipid-A in the inhibitory effect of LPS.

LPS-depleted fractions of Kpn KPC ST258 BE also lost their PMN stimulating properties, measured by the increase in the % of FSC and CD11b, whereas Kpn KPC ST258 BE+PMX directly added to PMN abolished the capacity of BE to induce an increase in the FSC and CD11b expression (Figures 8B,C).

Altogether, these results account for the polysaccharide part of LPS as responsible for the inhibition of fMLP-induced ROS.

## Discussion

Infections caused by KPC-producing Kpn ST258 are a major cause of healthcare-associated infections worldwide, including ventilator-associated pneumonia and catheter-related bloodstream infections (28, 29), and have been associated with increased cost and length of stay as well as frequent treatment failures and death (30–32). KPC producers are multidrug resistant (especially to all β-lactams), and therapeutic options for treating KPC-related infections remain limited. In parallel with their adaptation to antimicrobial exposure, some studies have demonstrated that these microorganisms have evolved mechanisms to evade host innate immune clearance. Evasion strategies are dangerous as persistence and chronic colonization not only select for more fit organisms, as previously demonstrated (33), but also increase the potential for invasive infection and further development of antibiotic resistance, particularly in vulnerable patient populations. Considering the clinical relevance of Kpn KPC ST258, in this study, we were interested in analyzing the interaction of this clone of Kpn with human PMN, a key cell of the innate immune response, in an effort to better understand the infection biology of this pathogen, a necessary aspect for the potential design of new strategies to treat Kpn infections.

Our results indicate that Kpn KPC ST258 is a poor inducer of the main bactericidal responses of PMN, ROS generation, and NETs formation compared to another opportunistic Gram-negative bacillus, like *E. coli*. ST258 is the most common sequence type of antibiotic resistant Kpn in our country and others (4, 34). However, these bacteria readily undergo recombination events and have highly variable plasmid content, antimicrobial resistance patterns, and capsular composition (35). Although Kpn may be typically considered as a single entity, even among the ST258 strains very different patterns of infection are elicited in model systems (36). Therefore, it was important to analyze local clinical isolates of the ST258 that varied in different aspects (Supplementary Materials 1, 3). Our results indicate that the lack of ROS induction was present in all ST258 strains analyzed, including isolates obtained from distant geographical cities within our country; this mechanism has been present since the year 2008, and was not exclusive for KPC producers, indicating that the absence of ROS represents an advantageous mechanism for the ST258 independently of the antibiotic resistant. Moreover, it is possible that our results can be extended to other not-ST258, as the ATCC clone used in this study showed a similar evasion strategy.

The lack of ROS induction could be explained if Kpn KPC ST258 was not being ingested by PMN. However, our results showed that the percentage of PMN that have phagocytized Kpn KPC ST258 and the number of Kpn KPC ST258 internalized *per* PMN were similar compared to Eco. In this regard, the amount of bacteria internalized *per* PMN is in line with the results shown by Kobayashi et al., who reported similar values for another ST258 strain (37). We have chosen *Eco* for comparison, as this bacterium is also a Gram negative bacillus from the *Enterobacteriaceae* family, with a similar size as Kpn, and therefore a good reference for comparison. Even though Kpn is considered as an example of a poor phagocytized organism, the percentages of phagocytosis of Kpn throughout the literature are variable and depend on the particular bacterial strain (38). However, what is consistent is that the amount of capsule (associated to hyermucoviscocity phenotypes) is crucial for being phagocytized and is usually correlated with resistance to complement killing (7). Our local strain ST258 of Kpn is not resistant to complement killing (our unpublished results) and is not phenotypically a mucoid strain, two characteristics that are aligned with a bacterium that is capable of being phagocytized.

What is important to highlight is that although phagocytosis was similar for both Kpn and Eco, Eco was able to trigger a high respiratory burst and Kpn KPC ST258 was not. Moreover, when we measured the overall result of PMN-Kpn interaction by measuring the remaining colony formation units (CFU), Kpn showed a higher survival compared to Eco. These results can be explained by different possibilities. It is possible, that Kpn is less sensitive to non-oxidative killing, or may be impairing mobilization to the phagosome of different NADPH subunits, as reported for other bacteria (39). We are currently investigating which of these mechanisms could be operating in Kpn KPC ST258-mediated ROS inhibition.

The widely held belief is that Kpn is a stealth pathogen, which fails to stimulate innate immune responses (40), shielding its pathogen-associated molecular patterns from detection by the immune system, thereby avoiding the interaction with hematopoietic and non-hematopoietic cells to prevent the activation of host antimicrobial responses. This belief is in contrast with our results that have shown that Kpn is being sensed by PMN, at least similarly to Eco, but for Kpn this leads to a lack of ROS induction. Additionally, our work described for the first time that Kpn KPC ST258 was able to modulate ROS triggered by Eco, indicating that the interaction of Kpn KPC ST258 with PMN was, in fact, negatively regulating ROS generation. This may also have clinical implications as the presence of Kpn may favor the survival and dissemination of other bacteria in cases of co-infections in hospitalized patients (41, 42).

Using BE we have demonstrated that Kpn was actively subverting host defenses. To evidence the absence of vs. an inhibitory phenomenon, a positive inducer, as was fMLP in our work, had to be used. Using this experimental approach, the inhibitory effect on ROS production mediated by a structural bacterial wall component of Kpn KPC ST258 was revealed. This was not surprising as capsule components or LPS have been reported as the responsible mediators of several evasion mechanisms of Kpn (43). In an attempt to elucidate the intracellular pathway affected by Kpn KPC ST258 in the cascade of events triggered by the ROS inducer fMLP, we performed several experiments using BE but, unfortunately, our results were not conclusive in this way, as Ca^2+^mobilization or NADPH oxidase assembly were not affected by the presence of BE. However, BE-mediated inhibition of ROS generation was also found for LB_4_, another classical ROS inducer that interacts with a seven-transmembrane receptor, indicating that Kpn KPC ST258 BE may be interfering with a common downstream pathway of this type of receptors.

LPS is a major component of the bacterial wall of Gram negative organisms. LPS consists of three different parts, in the following order, from inside the membrane to the outside: lipid A (also known as endotoxin), a core sugar consisting of 3-deoxy-D-manno-oct-2-ulosonic acid (Kdo) moieties, and the O-antigen, which consists of repeating oligosaccharide units. The lipid A component of LPS is recognized by Toll-like receptor 4 (TLR4) and its co-receptor MD-2 on host cells (44). Carbohydrates are usually recognized by other type of receptors, e.g., C-type lectin receptors, sometimes associated to inhibitory functions (45). Inhibitory receptors play key roles in regulating aspects of the immune response mainly by blocking activating pathways (46). Several inhibitory receptors have been described in PMN and are usually composed of a cytoplasmic tail that contains at least one immune-receptor tyrosine-based inhibitory motif (ITIM) (47). Some of these receptors have been shown to bind a diverse array of glycan ligands of endogenous or microbial origin. Our experiments using LPS-depleted and purified LPS clearly demonstrated that ROS inhibition triggered by fMLP was mediated by the LPS molecule of Kpn KPC ST258, and more specifically, by the polysaccharide portion. Moreover, LPS seems to play a dual role in its interaction with PMN. Whereas, the polysaccharide part of the molecule was found to be involved in ROS inhibition, neutralization of lipid A by PMX in the BE annulled the stimulation of cell size increase and CD11b expression, indicating that these effects were dependent on lipid A of Kpn's LPS. This is an interesting observation, as the same molecule can exert selectivity in its suppressive properties, avoiding bactericidal functions that are critical for bacterial survival. A certain functional selectivity was also found with the entire bacteria, which was able to affect specifically ROS and NETs generation but did not affect the increase in FSC and CD11b up-regulation. It should be noted that ROS inhibition mediated by LPS was partial, although the lack of induction caused by the whole bacteria was absolute. This indicates either that the native conformation of LPS could be more efficient compared to its form adopted after partial purification, or it could also be possible that more than one evasive molecule/strategy may be coexisting in Kpn.

Considering all this, it can be speculated that LPS can be delivering positive signals via TLR4 receptors by the lipid A portion (48), and, at the same time, negative signals through its interaction with another yet-unidentified receptor by the polysaccharide part. Supporting our findings of Kpn delivering negative signals, other authors have found that LPS O-polysaccharide of Kpn, although not ST258, abrogates the activation of inflammatory responses in the human lung alveolar epithelial A549 cells line by targeting NF-κB and MAPK signaling pathways (49, 50). We are currently investigating the identity of the putative inhibitory receptor involved in the phenomenon described in this work, as we believe that targeting inhibitory receptors that regulate the threshold activation of PMN would be beneficial in other clinical scenarios, as for the treatment of some inflammatory diseases.

In summary, our work describes new insights in the biology of Kpn KPC ST258, revealing active mechanisms of innate immune evasion. Moreover, the relevance of our findings can be extended to other pathophysiological contexts, but specifically, for Kpn infections, understanding the pathways involved in Kpn KPC ST258-mediated inhibition of ROS might provide the basis to delineate selective complementary alternatives for the management of this wide-spread, multi-resistant pathogen.

## Ethics Statement

Human normal samples were obtained from voluntary donors. This study was performed according to institutional guidelines (National Academy of Medicine, Buenos Aires, Argentina) and received the approval of the institutional ethics committee and written informed consent was provided by all the subjects.

## Author Contributions

LC, GF, VL, and SG conceived and designed the experiments. LC, FB-W, NR-R, and FB performed the experiments. LC, VL, DM-G, and GF analyzed and interpreted the data. GF and DM-G contributed with reagents, materials, and analysis tools. GF and LC wrote the manuscript. VL, DM-G, and SG revised the manuscript. LC, FB-W, NR-R, DM-G, FB-W, VL, SG, and GF approved the version to be published, and agreed to be accountable for all aspects of the work in ensuring that questions related to the accuracy or integrity of any part of the work are appropriately investigated and resolved.

### Conflict of Interest Statement

The authors declare that the research was conducted in the absence of any commercial or financial relationships that could be construed as a potential conflict of interest.
