# Supplementary material for: Klebsiella pneumoniae ST258 Negatively Regulates the Oxidative Burst in Human Neutrophils
Source: Front Immunol. 2019 Apr 26;10:929. doi: 10.3389/fimmu.2019.00929 (PMC6497972; doi:10.3389/fimmu.2019.00929)
Supplement: Supplementary file 1 [file Table_1.DOCX]

**Supplementary Material 1**

Most relevant characteristics of the different Kpn strains used to stimulate PMN

| **Strain ID** | **MLST** | **Location** |  | **Diagnostic** | **Isolation Year** | **Antimicrobial Resistance Mechanism** Φ |
| --- | --- | --- | --- | --- | --- | --- |
| **Kpn KPC ST258 (M9885)** | ST258 | CABA | Abdominal Abscess | Surgical Site Infection | 2008 | KPC-2 |
| **700603**  **(ATCC) #** | ST498 | - | - | - | - | *aadB*  *sul1*  SHV-18 |
| **M19216** | ST258 | CABA | Anal Swab | Carrier Screening | 2015 | CTX-M |
| **M19145** | ST258 | CABA | Anal Swab | Carrier Screening | 2015 | CTX-M |
| **M19091** | ST258 | Unknown | Anal Swab | Carrier Screening | 2015 | AMP-C |
| **M22738** | ST258 | Neuquén | Skin infection | Soft Tissue Infection | 2017 | KPC-2 |
| **M22810** | ST258 | Tucumán | Pleural Effusion | VAP* | 2017 | KPC-2 |
| **M22910** | ST258 | Salta | Sputum | Pneumonia | 2017 | KPC-2 |

* Ventilator- associated pneumonia

# <https://www.atcc.org/products/all/700603.aspx#generalinformation>

Φ Abbreviations: *aadB* (aminoglycoside), *sul1* (sulfonamide); ESBL (Extended-spectrum β-lactamases) encoding genes *bla*SHV18.CTX-M: plasmidic type, ESBL type; AMP-C: inducible ESBL type; KPC-2: carbapenemase type 2.
