# Supplementary figures and images for: Klebsiella pneumoniae ST258 Negatively Regulates the Oxidative Burst in Human Neutrophils
Source: Front Immunol. 2019 Apr 26;10:929. doi: 10.3389/fimmu.2019.00929 (PMC6497972; doi:10.3389/fimmu.2019.00929)

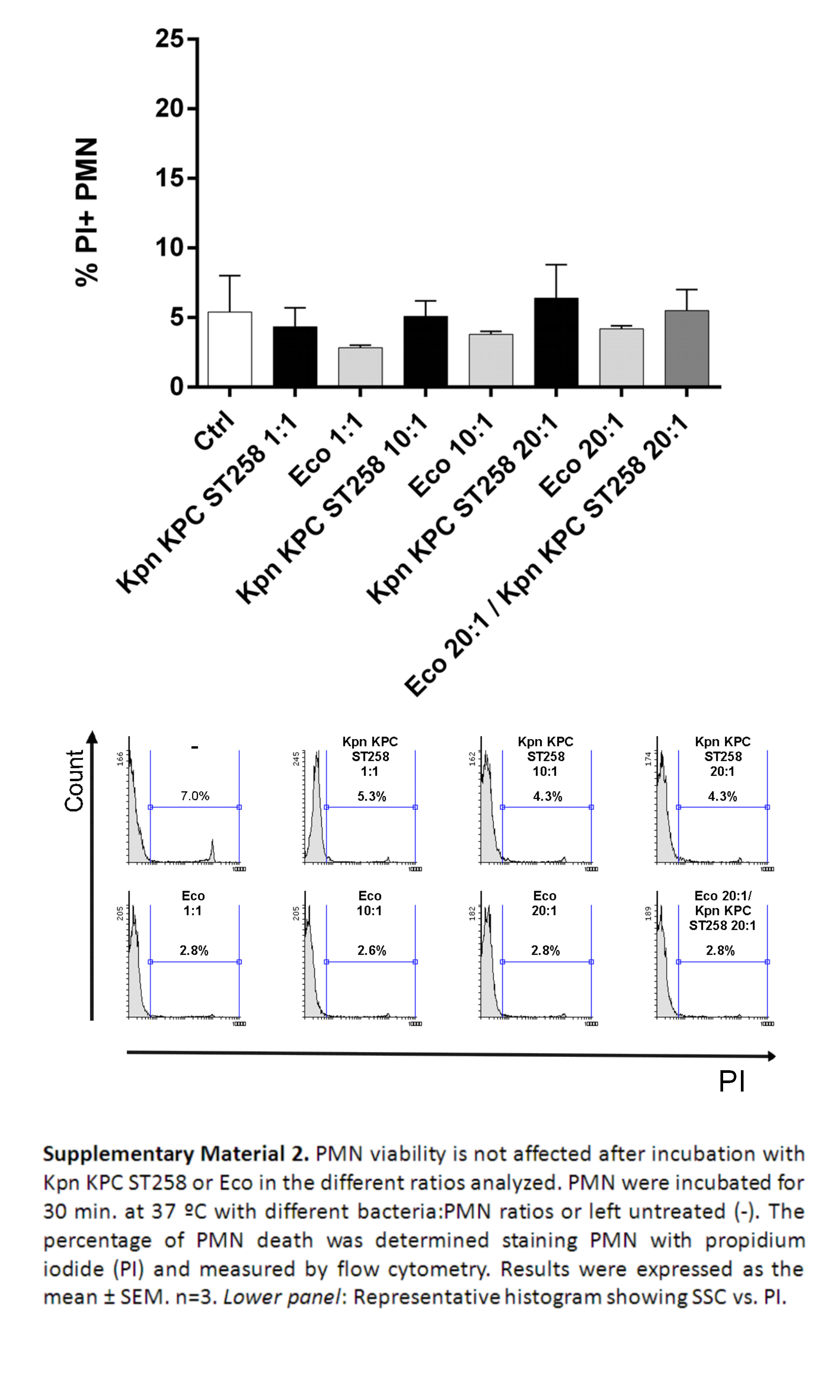

Supplement: Supplementary file 2 [file Image_1.tif]

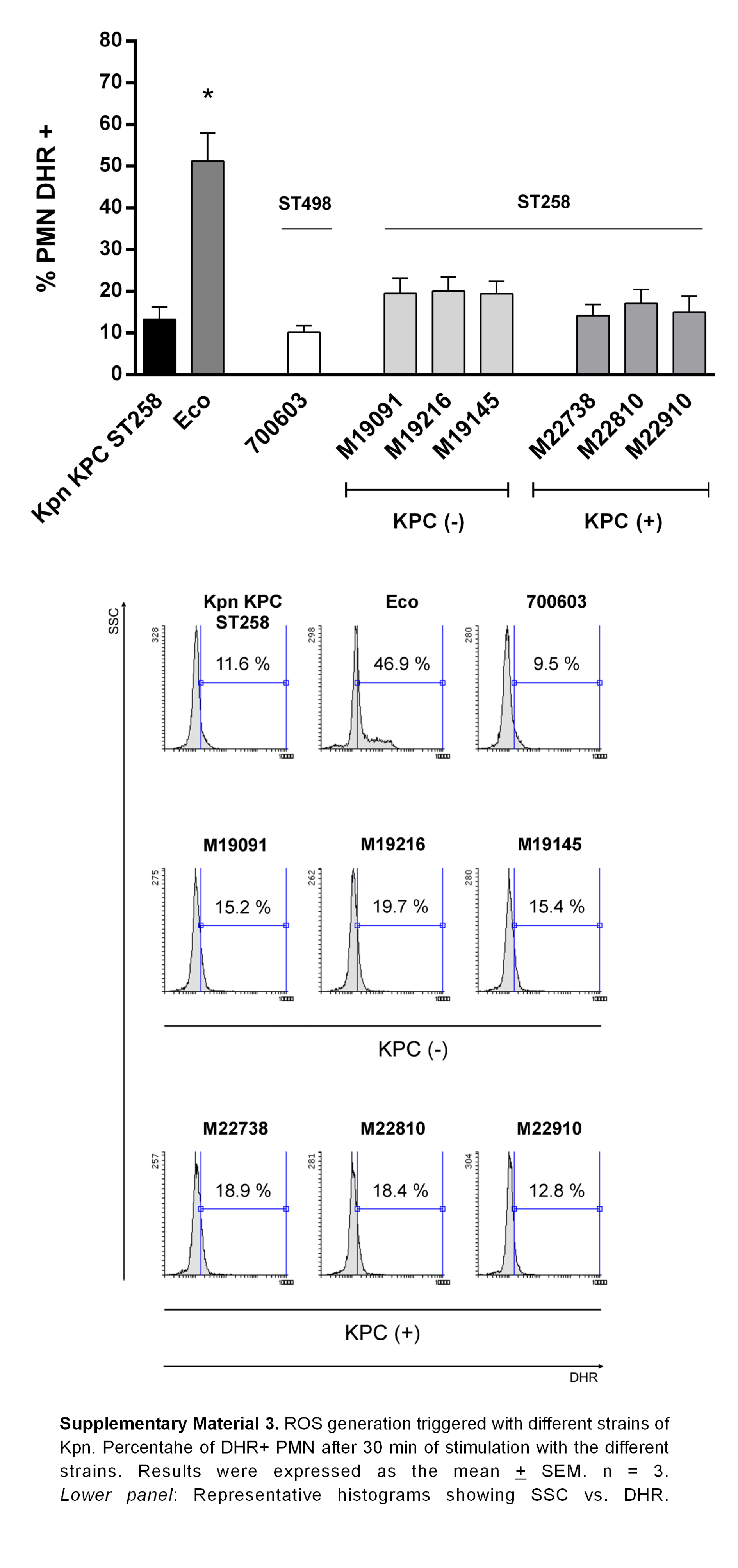

Supplement: Supplementary file 3 [file Image_2.tif]

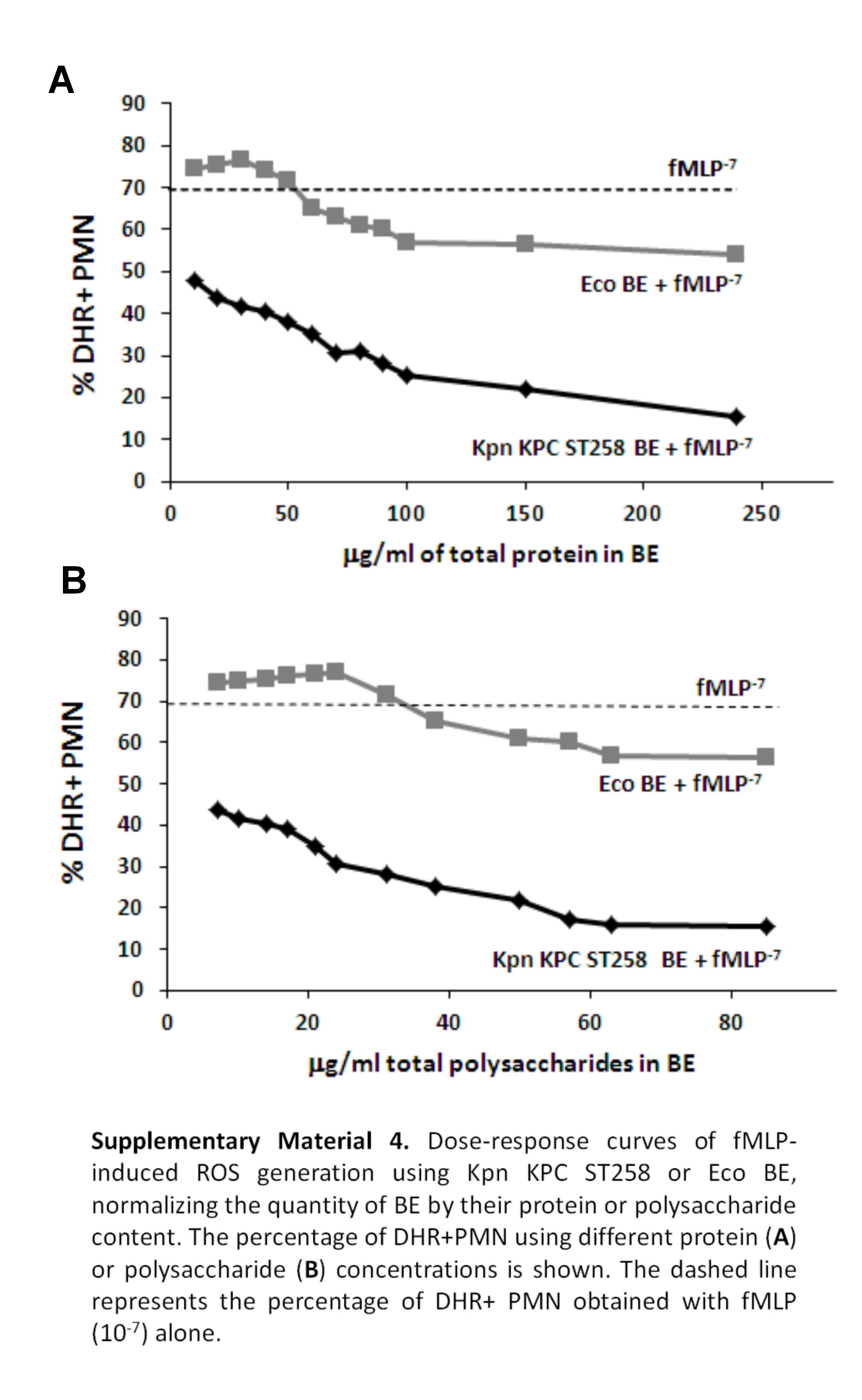

Supplement: Supplementary file 4 [file Image_3.tif]
